# Molecular Signaling Effects behind the Spontaneous Soleus Muscle Activity Induced by 7-Day Rat Hindlimb Suspension

**DOI:** 10.3390/ijms25158316

**Published:** 2024-07-30

**Authors:** Xenia V. Sergeeva, Kristina A. Sharlo, Sergey A. Tyganov, Vitaliy E. Kalashnikov, Boris S. Shenkman

**Affiliations:** Myology Laboratory, Institute of Biomedical Problems, RAS (Russian Academy of Sciences), Moscow 123007, Russia; sergeeva_xenia@mail.ru (X.V.S.); sentackle@yandex.ru (S.A.T.); vitaliy.kalasxnikov@yandex.ru (V.E.K.)

**Keywords:** soleus muscle unloading, muscle atrophy, KCC2

## Abstract

The elimination of ground reaction force (support withdrawal) vastly affects slow postural muscles in terms of their regulation and structure. One of the effects of support withdrawal in this study was an immediate postural muscle inactivation, followed by the daily gradual development of spontaneous activity of the slow postural soleus muscle in response to rat hindlimb suspension to mimic space flight. The origin of this activity is somewhat akin to muscle spasticity after spinal cord injuries and is the result of KCC2 content decline in the spinal cord’s motor neurons. However, the physiological consequences of unloading-induced spontaneous activity remain unexplored. We have conducted an experiment with the administration of a highly specific KCC2 activator during 7-day unloading. For this experiment, 32 male Wistar rats were divided into 4 groups: C+placebo, C+CLP-290 (100 mg/kg b w), 7HS+placebo, and 7HS+CLP—hindlimb-suspended group with CLP-290 administration (100 mg/kg b w). The soleus muscles of the animals were dissected and analyzed for several proteostasis- and metabolism-related parameters. CLP-290 administration to the unloaded animals led to the upregulation of AMPK downstream (p-ACC) and mTOR targets (p-p70S6k and p-4E-BP) and an enhanced PGC1alpha decrease vs. the 7HS group, but neither prevented nor enhanced atrophy of the soleus muscle or myofiber CSA.

## 1. Introduction

The motor system of mammals has evolved under the dominant influence of gravitational forces. According to the ideas propounded by the school of Professor Inessa Kozlovskaya, the stable position of the body and the locomotion of animals on the ground are ensured by the existence of two functional components of the motor system: tonic (slow) and phasic (fast). One of the key gravity-dependent functions of the motor system is to maintain a vertical body position in a gravitational field. These functions are provided by the tonic muscle system. Tonic movements are normally performed by special musculature, which is represented by the so-called slow/small motor units (MU) and slow muscle fibers.

In most mammals, the main slow muscle performing an antigravity function is the soleus muscle (*m. soleus*). It contains 70 to 90% of slow-type muscle fibers. The soleus muscle works at least 11 h a day [1]. The physiological consequences of eliminating the axial mechanical load and, especially, the ground reaction force for the soleus muscle include vast alterations in all the aspects of muscle functioning. Elimination of the ground reaction force is observed during space flight, as well as in ground-based models of microgravity effects, which include bed rest and “dry” immersion in humans and rodent hindlimb unloading.

In hindlimb unloading experiments in rats, zero-gravity parabolic flights, and “dry” immersion in humans, a dramatic decrease (sometimes up to complete suppression) in soleus muscle electrical activity has been repeatedly shown [2,3,4]. However, after the first several days of complete inactivation, soleus muscle EMG (electromyography) activity gradually increases during the course of unloading and, by the 14th day of hindlimb suspension (HS), reaches values comparable to those of the control animals [4]. This phenomenon was observed in rodent hindlimb unloading experiments simulating the effect of microgravity, in which soleus muscle EMG activity was recorded with the help of implanted electrodes.

However, the mechanisms of this spontaneous activity are still left unexplored. The physiological significance and consequences of this phenomenon are also currently unclear.

The mechanisms of spontaneous activity under skeletal muscle unloading conditions are somewhat similar to the molecular origin of muscle spasticity after spinal cord injuries. Briefly, a decrease in potassium (K^+^)/chloride (Cl^−^) co-transporter 2 (KCC2) expression due to spinal cord injury results in a more positive equilibrium potential for Cl^−^, with a subsequent change in synaptic input that would normally be inhibitory to an input with a more excitatory effect [5,6]. The inhibitory effects of GABA and glycine become more excitatory due to a positive shift in the equilibrium potential of Cl^−^ [6]. This switch can lead to hyperactivity in motoneurons and also spasticity [6]. It was found that increased excitability and activity of motoneurons due to KCC2 downregulation were also observed following peripheral axotomy [7]. In 2021, it was shown that the administration of KCC2 activator prochlorperazine during 7-day rat hindlimb suspension prevents the unloading-induced spontaneous activity of the soleus muscle [8].

The physiological significance of the development of unloading-induced spontaneous activity remains largely unexplored. It could either play a compensatory role, mitigating unloading-induced atrophy, or enhance atrophy via activity-dependent catabolic pathway activation. Prochlorperazine administration was shown to have some positive effects on the soleus muscle’s slow-to-fast fiber-type shift [9]. At the same time, prochlorperazine has a low specificity and has several adverse effects, including alpha-2-adrenoreceptors and D2 dopamine receptor-binding [10,11]. Based on these data, we decided to analyze the effects of CLP-290 administration on anabolic and catabolic signaling pathways in the soleus muscle during seven days of rat hindlimb unloading. CLP-290 is a novel specific KCC2 activator [12], so the effects of CLP-290 would be highly likely to be the result of KCC2 activation and unloading-induced spontaneous activity prevention.

## 2. Results

### CLP-290 Affects Soleus Muscle EMG Activity and KCC2 Content in the Spinal Cord Tissue

**Figure 1:**
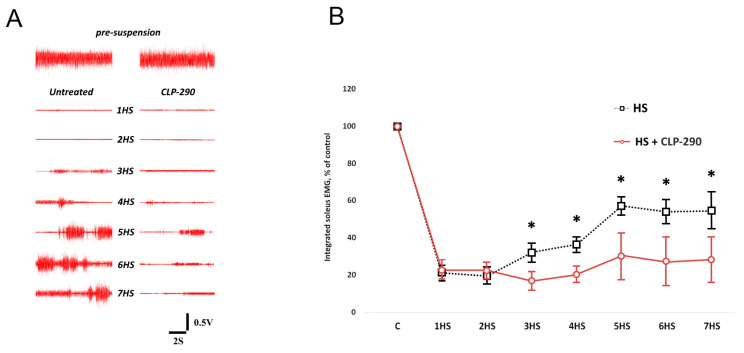
An example of a soleus muscle EMG in the experimental groups (**A**). EMG time-course for hindlimb-suspended rats with either the placebo or CLP-290 administration (**B**). *—significant differences from the HS group.

From day 1 to day 7 of the rat hindlimb suspension experiment, the EMG activity of the soleus muscles of the experimental animals in the 7HS group gradually increased after the significant decline of the soleus EMG compared with the C group on day 1 (Figure 1). In the 7HS CLP-290-administered group, the EMG activity of the soleus muscles was significantly lower than that in the HS group from day 3 to day 7 of the experiment.

**Figure 2:**
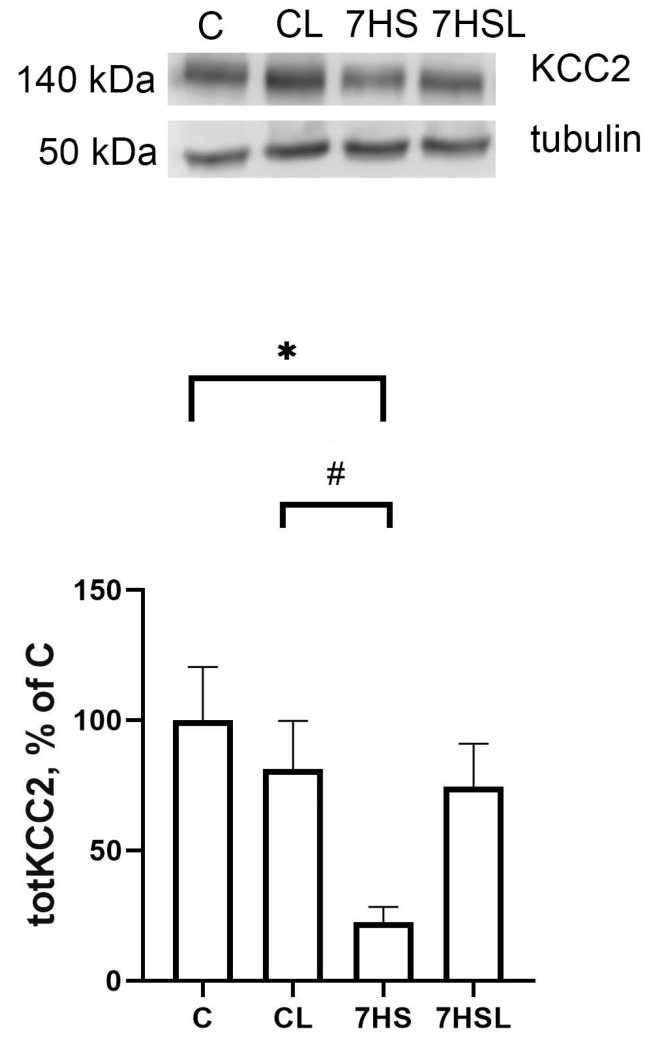
KCC2 content in the spinal cord tissue samples of the experimental animals. The data are represented as a percentage of the C-group mean value ± SEM. C—vivarium control, CL—control group with CLP-290, 7HS—7-day hindlimb-suspended group, 7HSL—7-day hindlimb-suspended group with CLP-290. *—significant differences from the C group; #—significant differences from the CL group.

The content of KCC2 in the spinal cord tissue samples of the experimental animals did not differ between the C and CL groups, while in the 7HS group, the KCC2 content was significantly lower compared with the C and CL groups. The content of KCC2 in the 7HSL group did not significantly differ from any other experimental group (Figure 2).

**Figure 3:**
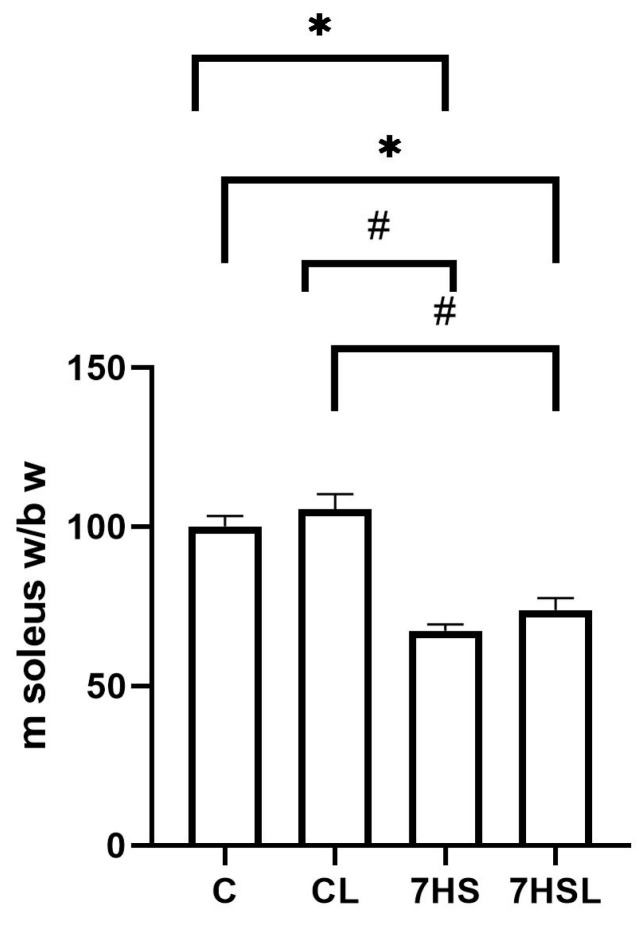
Soleus muscle weight, normalized to the body weight of the experimental animals. The data are represented as a percentage of the C group mean value ± SEM. C—vivarium control, CL—control group with CLP-290, 7HS—7-day hindlimb-suspended group, 7HSL—7-day hindlimb-suspended group with CLP-290. *—significant differences from the C group; #—significant differences from the CL group.

Soleus muscle weight, when normalized to animal body weight, did not differ between the C and CL groups, while in both hindlimb-suspended groups, the normalized soleus muscle weight revealed a significant reduction compared to both control groups. The 7HS and 7HSL group values did not differ from each other (Figure 3).

**Figure 4:**
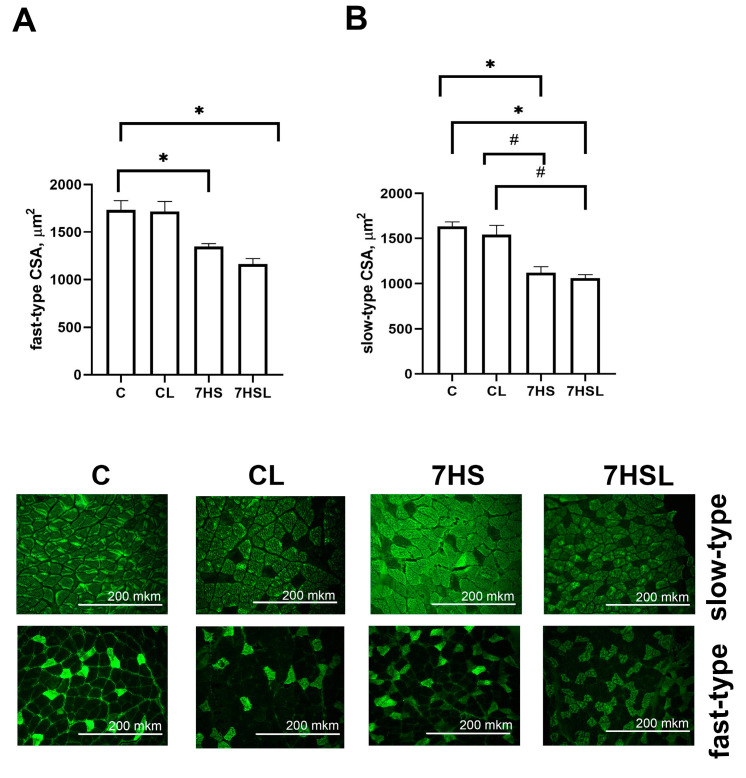
The fast-type muscle fiber cross-sectional area (CSA) (**A**) and slow-type muscle fiber CSA (**B**) of the soleus muscles of the experimental animals. The panel below shows microphotographs of the soleus muscles’ cross-sectional area, immunostained against slow-type or fast-type myosins. The data are represented as mean values ± SEM. C—vivarium control, CL—control group with CLP-290, 7HS—7-day hindlimb-suspended group, 7HSL—7-day hindlimb-suspended group with CLP-290. *—significant differences from the C group; #—significant differences from the CL group.

No differences were detected in either the fast-type or slow-type CSA between the C and CL experimental groups (Figure 4). At the same time, in both hindlimb-suspended groups, the slow-type and fast-type CSAs were significantly lower than in the C and CL groups, while there were no differences between the 7HS and 7HSL groups (Figure 4).

**Figure 5:**
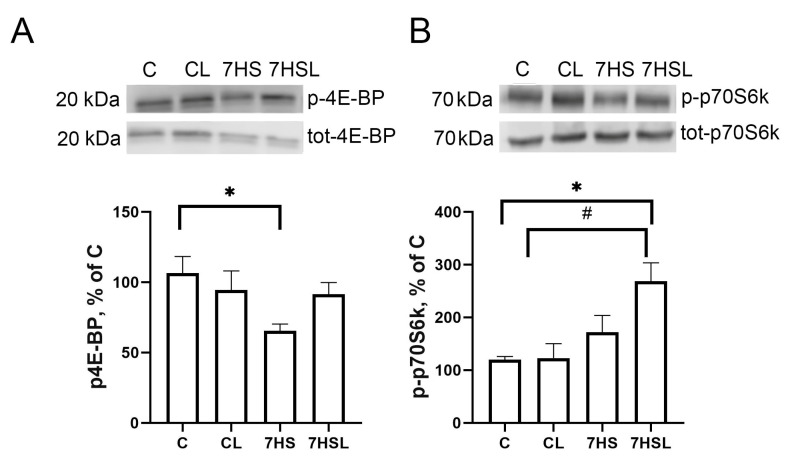
The p-4E-BP (**A**) and p-p70S6k (**B**) contents in the soleus muscle protein samples from the experimental animals. The data are represented as a percentage of the C group mean value ± SEM. C—vivarium control, CL—control group with CLP-290, 7HS—7-day hindlimb-suspended group, 7HSL—7-day hindlimb-suspended group with CLP-290. *—significant differences from the C group; #—significant differences from the CL group.

The contents of p-4E-BP and p-p70s6k, which are both mTOR downstream targets, did not differ between the C and CL groups (Figure 5). In the 7HS group, the content of p-4E-BP was significantly lower than in the C group, while the content of p-p70S6k did not differ from both the control groups, C and CL. The content of p-4E-BP in the 7HSL group did not differ from any other experimental group, and the content of p-p70S6k in the 7HSL group was significantly higher than in the C and CL groups (Figure 5).

**Figure 6:**
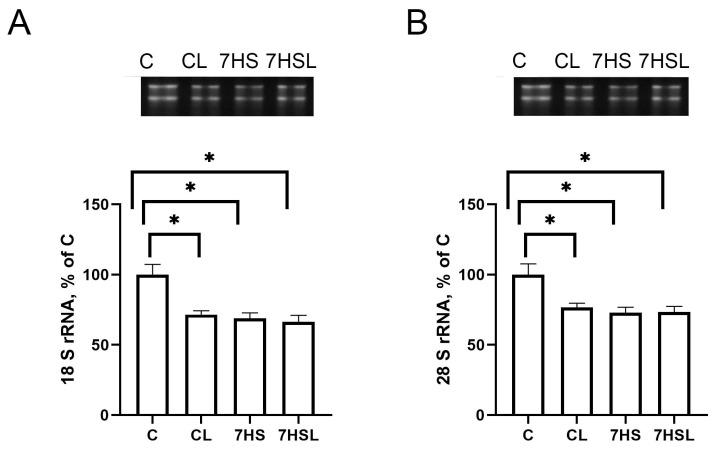
Contents of 18S rRNA (**A**) and 28S rRNA (**B**) in the soleus muscles of the experimental animals. The data are represented as a percentage of the C group mean value ± SEM. C—vivarium control, CL—control group with CLP-290, 7HS—7-day hindlimb-suspended group, 7HSL—7-day hindlimb-suspended group with CLP-290. *—significant differences from the C group.

The contents of both the 18S rRNA and 28S rRNA were significantly lower in the CL, 7HS, and 7HSL groups compared to the C group, while the rRNA content did not differ among the three mentioned groups (Figure 6A,B).

**Figure 7:**
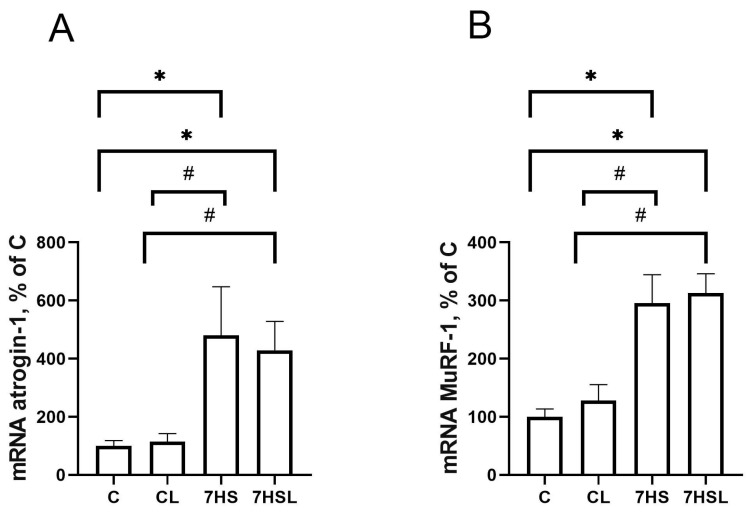
Atrogin-1 mRNA expression (**A**) and MuRF-1 mRNA expression (**B**) in the soleus muscles of the experimental animals. The data are represented as a percentage of the C group mean value ± SEM. C—vivarium control, CL—control group with CLP-290, 7HS—7-day hindlimb-suspended group, 7HSL—7-day hindlimb-suspended group with CLP-290. *—significant differences from the C group; #—significant differences from the CL group.

The mRNA expression of the key E3 ubiquitin ligases mediating muscle proteolysis, MuRF-1 and Atrogin-1, did not differ between the C and CL groups, while in both hindlimb-suspended groups, their mRNA expression was significantly higher than in the control groups (Figure 7). Both the MuRF-1 and Atrogin-1 mRNA expression levels did not differ between both suspension groups of 7HS and 7HSL (Figure 7A,B).

**Figure 8:**
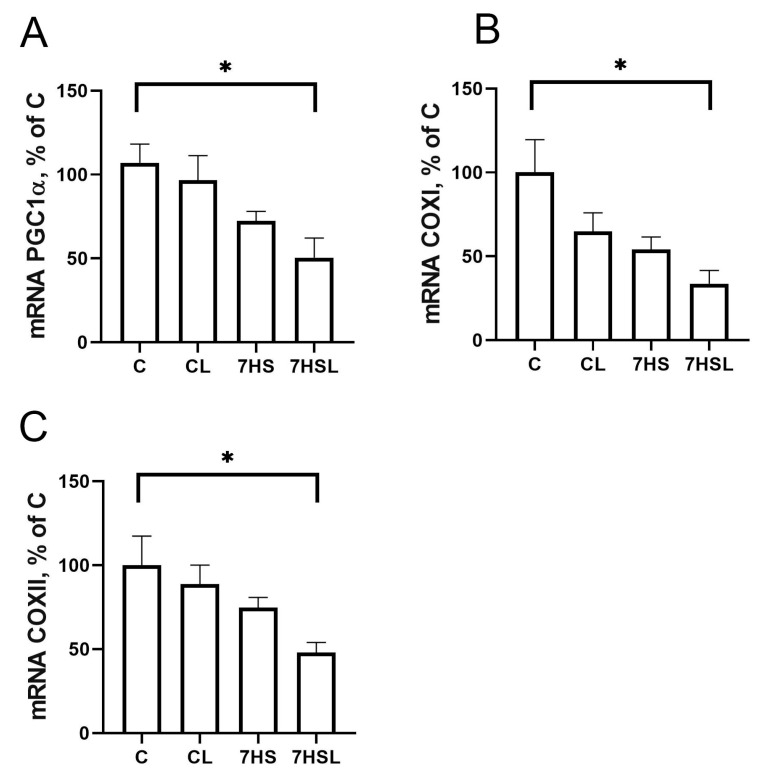
PGC1α mRNA expression (**A**), COXI mRNA expression (**B**), and COXII mRNA expression (**C**) in the soleus muscles of the experimental animals. The data are represented as a percentage of C group mean value ± SEM. C—vivarium control, CL—control group with CLP-290, 7HS—7-day hindlimb-suspended group, 7HSL—7-day hindlimb-suspended group with CLP-290. *—significant differences from the C group.

There were no significant differences in the mRNA expression of the mitochondrial biogenesis regulator PGC1α and its downstream targets, COXI and COXII, across the experimental groups, except for the significant downregulation of all three parameters in the 7HSL group compared with the C group (Figure 8A–C).

**Figure 9:**
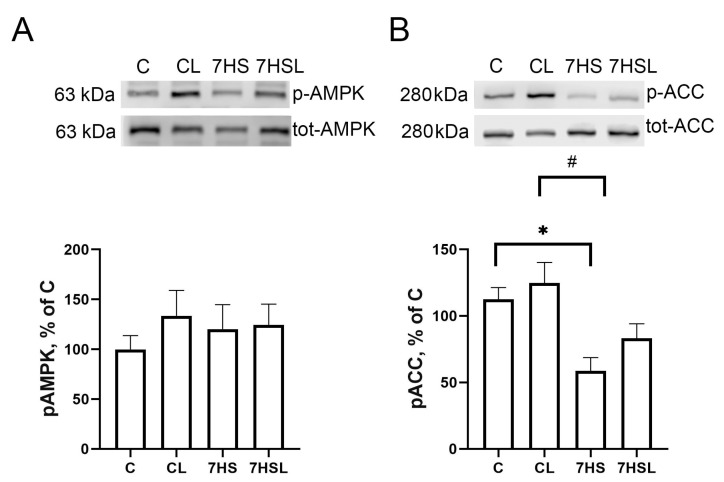
pAMPK content (**A**) and pACC content (**B**). The data are represented as a percentage of the C group mean value ± SEM. C—vivarium control, CL—control group with CLP-290, 7HS—7-day hindlimb-suspended group, 7HSL—7-day hindlimb-suspended group with CLP-290. *—significant differences from the C group; #—significant differences from the CL group.

There were no significant differences in the p-AMPK content values across the experimental groups (Figure 9A). The content of the AMPK target p-ACC was significantly lower in the 7HS group compared to the C and CL groups, while none of the other groups differed significantly (Figure 9B).

**Figure 10:**
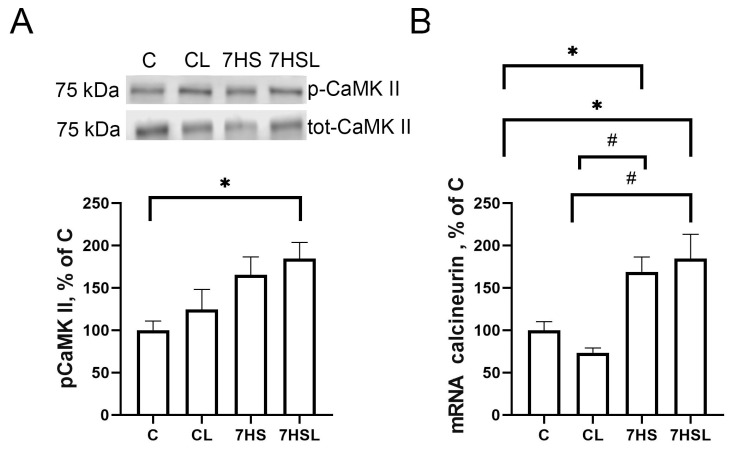
pCaMK II content (**A**) and calcineurin A mRNA expression (**B**). The data are represented as a percentage of the C group mean value ± SEM. C—vivarium control, CL—control group with CLP-290, 7HS—7-day hindlimb-suspended group, 7HSL—7-day hindlimb-suspended group with CLP-290. *—significant differences from the C group; #—significant differences from the CL group.

The content of p-CaMK II did not differ among the experimental groups, except for its significantly higher content in the 7HSL group compared to the C group (Figure 10A). The mRNA expression of calcineurin A was significantly higher in the 7HS and 7HSL groups compared to the C and CL groups, while the C and CL groups did not differ from one another, and neither did the 7HS and 7HSL groups (Figure 10B).

**Figure 11:**
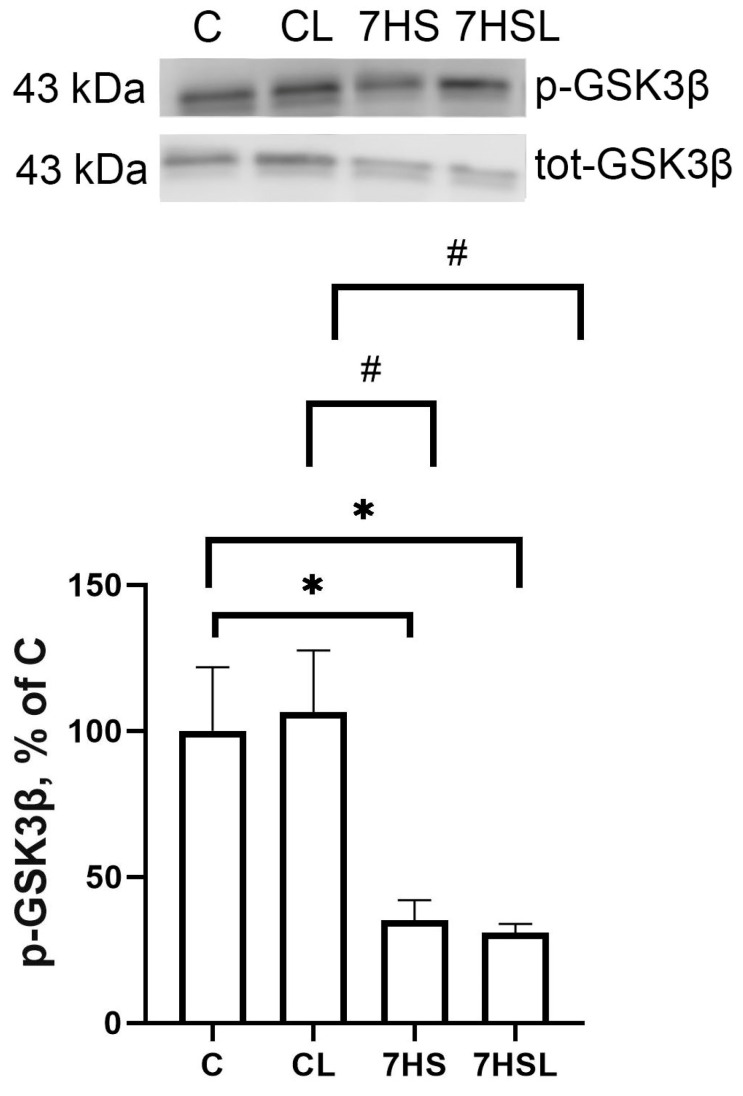
p-GSK3β content. The data are represented as a percentage of the C group mean value ± SEM. C—vivarium control, CL—control group with CLP-290, 7HS—7-day hindlimb-suspended group, 7HSL—7-day hindlimb-suspended group with CLP-290. *—significant differences from the C group; #—significant differences from the CL group.

The content of p-GSK3β did not differ between the C and CL groups, while in both hindlimb-suspended groups, the content was significantly lower compared to both control groups. The 7HS and 7HSL groups did not differ from one another (Figure 11).

## 3. Discussion

After 7 days of hindlimb suspension, we detected the key signaling changes that are characteristic of this time-point for the model: the atrophy of both slow-type and fast-type fibers; the upregulation of proteolysis markers; a decrease in protein synthesis markers; a decline in soleus muscle weight. These data correspond to the previous experiments [13,14].

The effects of CLP-290 on soleus muscle EMG activity, as well as its effect on KCC2 content in the spinal cord tissue of the hindlimb-suspended animals, are in good agreement with the previously published effects of prochlorperazine on these parameters [8]. However, a number of signaling effects that were observed in the prochlorperazine-administered hindlimb-suspended animals in [9] were not detected in the current study.

Summing the effects of the two substances blocking the spontaneous electrical activity of the soleus muscles in hindlimb-unloaded animals, we can divide them into two groups: effects common to both prochlorperazine and CLP-290, and effects that are unique to prochlorperazine. It would be reasonable to assume that the similar effects of prochlorperazine and CLP-290 are caused by KCC2 activation, leading to spontaneous activity inhibition. These effects are an increase in the phosphorylated contents of p70S6 kinase, 4E-BP, and ACC, and the downregulation of PGC1α, COXI, and COXII mRNA expression when compared to the 7HS group [9,15].

Phosphorylated ACC content is a marker of AMPK (AMP-activated protein kinase) activity, as the Ser 79 residue of ACC is a direct downstream target of AMPK. AMPK, in turn, was shown to be activated by an ATP/ADP ratio decrease and glycogen depletion, which are usually the result of long-term muscle activity [16]. At the same time, AMPK is an activator of mitochondrial biogenesis, including PGC1α mRNA expression. After 7 days of hindlimb suspension, we did not detect any changes in p-AMPK content, but the p-ACC content, which is a marker of AMPK activity, was downregulated. This observation agrees well with other studies reporting findings at the 7-day hindlimb suspension time point [17].

In the current study, we can observe a paradoxical situation: blocking muscle activity (albeit a specific type of activity) leads to the upregulation of a marker of AMPK activity, which is accompanied by the enhanced downregulation of mRNA expression of the key mitochondrial biogenesis regulator, PGC1α.

This paradox can be resolved if we assume that PGC1α downregulation could be the direct effect of blocking spontaneous muscle activity. It should be noted that PGC1α downregulation is accompanied by AMPK activation in the CLP-290-administered group. Therefore, this effect of CLP-290 seems not to be dependent on AMPK-mediated PGC1α regulation. Previously, it has been shown that PGC1α mRNA expression in the skeletal muscle is controlled by multiple activity-dependent signaling pathways. One of the activity-dependent signaling mechanisms could have been suppressed by CLP-290 blocking spontaneous soleus muscle activity, which could further inactivate PGC1α expression [18]. In turn, the downregulation of the key regulator of mitochondrial biogenesis could result in ATP depletion. In our previous article, we have shown that prochlorperazine administration during a 7-day hindlimb suspension test using rats led to ATP downregulation in soleus muscle tissue [9]. Based on this data, we can suggest that this ATP depletion could be the result of the suppression of mitochondrial biogenesis. This decline in ATP content may, in turn, be the reason for AMPK activation, leading to p-ACC accumulation in the soleus muscles of unloaded animals. However, the mechanism for mitochondrial biogenesis suppression by reducing soleus muscle EMG activity remains unknown.

Another common effect of the CLP-290 and prochlorperazine treatment of hindlimb-suspended animals is an increase in p-p70S6 kinase content compared with the 7HS group. The Thr-389 residue of p70 is a downstream target of mTOR protein kinase. Recently, it has been shown that ceramide accumulation in the skeletal muscle can enhance the Thr-389 phosphorylation of S6 kinase by activating mTOR [19]. Ceramide was shown to become accumulated after short-term disuse when EMG activity was almost completely eliminated and the p-p70S6 content was upregulated [14,20]. Previous studies have shown p-p70S6 kinase content upregulation in the soleus muscle after 1–3 days of rat hindlimb suspension when soleus muscle activity was eliminated and showed its gradual decline from day 7 to day 14, accompanied by the step-by-step development of spontaneous muscle activity [4,14,20].

Thus, we can suggest that CLP-290 and prochlorperazine treatment could enhance p-p70 content via EMG downregulation, which is possibly mediated by unloading-induced ceramide accumulation.

CLP-290 administration did not lead to the prevention of atrophy in soleus fibers, rRNA content increase, the attenuation of MuRF-1, and atrogin-1 upregulation, in contrast to the previously reported prochlorperazine effects [15]. Prochlorperazine is a less specific KCC2 activator than CLP-290, so it can be assumed that all the effects unique to prochlorperazine are mediated by some of its non-specific properties. In particular, prochlorperazine was shown to block alpha-1 adrenergic receptors, which could function as calcium channels when activated [10]. These receptors are usually described as being expressed in the smooth muscle cells, but there are some observations detecting the presence of alpha-1 adrenergic receptors in the skeletal myofibers of the soleus muscle [21]. The prochlorperazine-induced inhibition of alpha-1 adrenergic receptors could possibly affect the myoplasmic calcium content in soleus muscle fibers. Indeed, in contrast to the previously reported prochlorperazine effects, we did not detect any prevention of the unloading-induced p-CaMK II content or any calcineurin mRNA expression increase. These are both indicators of calcium-dependent signaling upregulation [9].

Excessive myoplasmic calcium accumulation was shown to contribute to MuRF-1 mRNA expression upregulation in the soleus muscle during unloading [22]. It could also contribute to the atrophy of skeletal muscle fibers via calpain activation [23]. Consequently, the effects of prochlorperazine on the atrophy of slow-type myofibers and the markers of proteolytic signaling could be the results of its effect on myoplasmic calcium content in the unloaded soleus muscles of the experimental animals. However, the mechanisms of the protective effects of prochlorperazine administration on rRNA content remain elusive, as there are no literature data showing any calcium-induced effects on rRNA content during rat hindlimb unloading. At the same time, we can assume that the effects of prochlorperazine on rRNA content are not mediated by its effect on delayed onset muscle activity, as CLP-290 had no effect on rRNA content.

**Figure 12:**
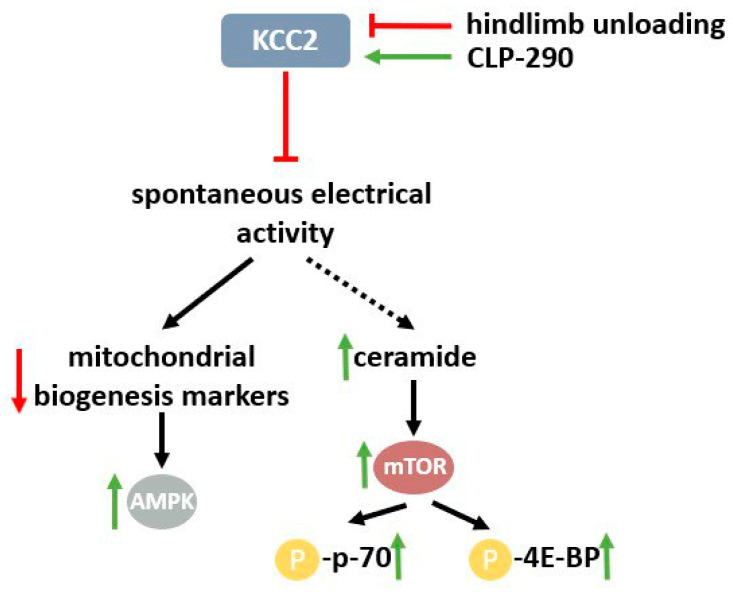
Signaling effects of CLP-290 in the unloaded soleus muscle. The red lines show inactivation, while the green lines show activation.

Altogether, we can conclude that the blocking of delayed-onset soleus muscle activity via KCC2 activation leads to the downregulation of AMPK downstream (p-ACC) and the upregulation of mTOR targets (p-p70S6k and p-4E-BP), but this neither prevents nor enhances atrophy of the soleus muscle or myofiber CSA. The signaling effects of CLP-290 in the unloaded soleus muscle are summarized in Figure 12.

## 4. Materials and Methods

### 4.1. Experimental Design

For this study, 32 male Wistar rats that were 1.5–2 months old and 180–200 g in body weight were randomly assigned into 4 groups (8 animals in each): C+placebo (15% cyclodextrin in saline), C+CLP-290 (100 mg/kg b w); 7HS+placebo, 7HS+CLP—hindlimb-suspended group with CLP-290 administration (100 mg/kg b w with 15% filtered cyclodextrin in saline). The substances and placebo were administered by intraperitoneal injections. All the experiments were approved by the Biomedicine Ethics Committee of the Institute of Biomedical Problems of the Russian Academy of Sciences (ethical statement number 638, 18 April 2023). Half of the animals from each group underwent electrode implantation for further EMG detection, as described below.

Hindlimb suspension was performed via a standard model protocol, which has been described elsewhere (Morey-Holton and Globus 2002) [13]. Briefly, a U-shaped wire fragment was fixed on the rat’s tail with a strip of adhesive tape. The wire was then attached to a swivel that freely moved along a metal bar on the top of the cage. The animals could move around the cage and had free access to food and water. The height of the suspension was adjusted to prevent the animals’ hindlimbs from touching any supporting surface, with the body of the animal suspended at a 45-degree angle to the cage floor. The weight of the experimental animal did not change significantly after the experiment, nor were there any significant differences in body weight among the experimental groups.

After the experiment, the animals were narcoticized with isoflurane, then both of their soleus muscles and lumbar spinal cord sections were dissected and immediately frozen in liquid nitrogen. After that, the narcoticized animals were sacrificed by dislocation of the neck.

### 4.2. Implantation of Electrodes into the Soleus Muscle

The electromyogram of the rat *m. soleus* was recorded using intramuscular electrodes. Teflon-coated stainless steel stranded wires (A-M Systems, Sequim, WA, USA) were used as electrodes. The insulation was removed from the wires over a 2-mm section, after which they were implanted into the soleus muscle of the right hindlimb of the rat, leading subcutaneously from the muscle to a socket located on the back of the rat. The sockets were made on a 3D printer. The wide brim of the rosette had holes for sewing it to the skin of the rat’s back. To protect the socket from possible damage while the rat was in the cage, a removable protective cap was made using 3D printing to cover the socket on the back.

The operation to implant electrodes into the rats’ *m. soleus* was performed under general anesthesia. Zoletil-100 was used at a dose of 28 mg/kg and given intramuscularly, along with xylazine hydrochloride (XylaVET 20 mg/mL) at a dose of 0.28 mL/kg body weight, also intramuscularly. The hair at the muscle access points was removed using a Codos veterinary trimmer. To access the *m. soleus*, an incision of about 1.5 cm was made on the lateral surface of the leg. To install the socket, an incision was made about 1.5 cm above the lumbar spine. Bleeding from the vessels of the skin of the back was stopped with an electrocoagulator. The wires were secured using 4-0 Ethylon knots above and below the muscle exit site. A rosette with a thin silicone oval sewn onto it was installed in a wound on the lower back and sewn to the skin with ethylene-4. The muscles and fascia on the leg were sutured with Vicryl (Vicryl 5-0, needle 11 mm, 3/8, reverse-cutting needle, W9501T, Ethicon, Raritan, NJ, USA), and the skin with Ethilon (Ethilon 4-0, needle 19 mm, 3/8, reverse cutting needle, W1619T, Ethicon, Raritan, NJ, USA). After the operation, the rat was subcutaneously injected with about 5 mL of saline and bicillin-3 at a single dose of 120,000 units/kg. The animal then recovered for 7 days before undergoing electrophysiological studies.

### 4.3. EMG Detection

The electromyographic signal was amplified using an AM-Systems 1700 differential AC amplifier with a sampling frequency of 5 kHz. The raw signal was filtered using low cut-off (100 Hz) and high cut-off (5000 Hz) filters. Control EMG values were recorded for 1 day, after which the animals were suspended. EMG recordings were carried out daily for 45 min. The received signal was processed using the ADC module L-CARD E14-440D and LGRAPH2 and the Powergraph 3.3 software. To evaluate the spectral characteristics of the received signal, a fast Fourier transform (FFT) technique was used, after which additional digital bandpass filters were applied. After this, the RMS envelope of the value module of the received signal was constructed and the integral of this envelope was calculated.

### 4.4. Protein Extraction and Western Blots

Frozen soleus muscle samples were cryosectioned on Leica cryostat (20 μm, 15–25 mg). The soleus cryosections or spinal cord samples were homogenized for 15 min in RIPA buffer (#sc-24948, Santa Cruz Biotechnology, Dallas, TX, USA) with additional proteinase and phosphatase inhibitors, using TissueLyser LT (QIAGEN, Hilden, Germany). After that, the samples were centrifuged for 15 min at 2000× *g*, then the supernatant was collected and stored at −80 °C.

A Bradford protein assay (Bio-Rad Laboratories, Hercules, CA, USA) and Epoch spectrophotometer (Bio-Tek Instruments, Winooski, VT, USA) were used to determine the protein concentration. The supernatant fluid was diluted with 2X Laemmli sample buffer (with an additional 10% of β-mercaptoethanol and 0.02% bromphenol blue) and stored at −80 °C for the immunoblot procedures.

For Western blot analysis, the samples that were diluted in Laemmli buffer were run on 10% SDS-PAGE (20 µg/lane) using mini-Protean 3 Cell (Bio-Rad Laboratories, Hercules, CA, USA). The proteins were transferred to a nitrocellulose membrane (Bio-Rad Laboratories, Hercules, CA, USA). SDS-PAGE analysis, followed by the Western blot procedure and analysis, were conducted as previously described in [24]. The primary antibodies used in this study were the total GSK3β and phosphorylated (Ser 9) GSK3β (Cell Signaling Technology, Danvers, MA, USA, 1:1000 # 9322, # 12456), phosphorylated (Ser 79) ACC (1:1000, Cell Signaling Technology, Danvers, MA, USA, #3661), total ACC (1:2000, Cell Signaling Technology, Danvers, MA, USA, #2072), total CaMK II (CSB-PA061493, Cusabio, Wuhan, China, 1:1000), phospho-CaMK II (CSB-PA283993, Cusabio, Wuhan, China, 1:1000), p-p70S6K (Thr 389) (1:500; Santa Cruz Biotechnology, Dallas, TX, USA, #sc-11759), p70S6K (1:1000, Cell Signaling Technology, Danvers, MA, USA, #9202), total KCC2 (1:1000, cat. #07-432; Merck, Burlington, MA, USA). Antibodies against GAPDH (1:10,000, G 041; ABM, Richmond, BC, Canada) were used for normalization in the muscle tissue, while antibodies against tubulin (cat. #ab176560; Abcam, Boston, MA, USA) were used for normalization in the nervous tissue.

The secondary antibodies used were horseradish peroxidase-conjugated goat anti-rabbit (1:30,000, #111-035-003, Jackson Immuno Research, West Grove, PA, USA) or goat anti-mouse (1:20,000, #1706516, Bio-Rad, Hercules, CA, USA).

### 4.5. Immunohistochemistry

Two series of soleus muscle samples from transverse frozen sections (8 μm) were prepared using a Leica CM 1900 cryostat (Braunschweig, Germany), then dried at room temperature for 15 min, and, finally, incubated in PBST for 20 min. Sections were incubated with primary antibodies (MyHC I(β) slow, 1:100, Sigma, St. Louis, MO, USA) or MyHC fast at 1:60, (DSMZ, Braunschweig, Germany) for 1 h at 37 °C. The anti-MyHCs fast antibody used in this study does not distinguish between the different fast MyHC isoforms. After three 10-min washes with PBST, the sections were incubated with secondary antibodies (Alexa Fluor 488, 1:1000; Molecular Probes, Waltham, MA, USA) for 60 min in the dark at room temperature. Subsequently, the sections were washed 3 times with PBST, examined, and photographed with a Leica Q500MC (Wetzlar, Germany) fluorescent microscope at a 20× objective magnification. The area of the myofibers was detected using ImageJ 4.0 software. At least 100 fibers of each type were evaluated in every sample. This number is sufficient to evaluate the myofibers’ characteristics.

### 4.6. RNA Extraction, rRNA Electrophoresis, and RT-PCR

RNA from the frozen samples was isolated with an RNeasy Micro Kit (Qiagen, Hilden, Germany) according to the recommendations of the manufacturer. The concentration of RNA in the samples was evaluated using a NanoPhotometer spectrophotometer (Implen GmbH, Munich, Germany). Reverse transcription was performed using an RT kit OT-1 (Syntol, Moscow, Russia) according to the manufacturer’s instructions. Then, 1 microgram of total RNA was used for the reverse transcription. Amplification was performed with a Quantitect SYBR Green Master Mix (Syntol, Moscow, Russia) and 10 pM of each forward and reverse primer in an iQ5 Multicolor Real-Time PCR detection system (Bio-Rad Laboratories, Hercules, CA, USA). The primer sequences are listed in Table 1. A melting curve analysis was used to verify the specificity of the primers. Relative quantification was performed using the Pfaffl method (32). RPL19 was chosen as a reference gene since its expression did not significantly differ across the groups.

For RNA electrophoresis, the RNA samples were diluted in 2× denaturing loading dye buffer (Thermo Fisher, Waltham, MA, USA), after which they were loaded onto a 1.2% agarose gel with the addition of ethidium bromide. The sample volume for loading was calculated based on the mass of muscle tissue from which the RNA sample was isolated. Electrophoresis was carried out at a voltage of 5 V per cm of the gel length in TBE buffer. RNA content was assessed using the optical density of the gel bands and was analyzed on a Gel Doc scanner (Bio-Rad, Hercules, CA, USA).

## Figures and Tables

**Table 1:** PCR primers used in the study.

MuRF-1	5′GCCAATTTGGTGCTTTTTGT3′ 5′AAATTCAGTCCTCTCCCCGT3′
Atrogin-1	5′CTACGATGTTGCAGCCAAGA3′ 5′GGCAGTCGAGAAGTCCAGTC3′
PGC1alpha	5′GTGCAGCCAAGACTCTGTATGG3′ 5′GTCCAGGTCATTCACATCAAGTTC3′
RPL19	5’GTACCCTTCCTCTTCCCTATGC3’ 5’CAATGCCAACTCTCGTCAACAG3’
COX II	5TGGCTTACAAGACGCCACAT-3’ 5-TGGGCGTCTATTGTGCTTGT-3’
COX I	5′ATTGGAGGCTTCGGGAACTG3′ 5′AGATAGAAGACACCCCGGCT3′

## Data Availability

The original contributions presented in the study are included in the article; further inquiries can be directed to the corresponding author/s.
